# Monitoring birds interacting with power lines: a systematic review of detection technologies and the persistent gap in transmission line applications

**DOI:** 10.1007/s10661-026-15647-w

**Published:** 2026-07-25

**Authors:** Ícaro Menezes Pinto, Luiggi Cavalcanti Pessôa, Pedro Ricardo Nery Nunes Silva, Gabriela Gama da Silva Santos, Viviane Spencer Andrade, Benito Rafael Santana de la Torre, Pedro Emanuel Santos Machado, Eduarda Silva Almeida, Larissa Donida Biasotto, Alessandra Schwertner Hoffmann, Michele Ferreira Lima, Ricardo Abranches Felix Cardoso Junior, Beatriz Cabral Dias de Carvalho

**Affiliations:** 1 Universidade SENAI CIMATEC - Centro Integrado de Manufatura e Tecnologia, Salvador, Bahia Brazil; 2 BirdLife International, Cambridge, UK; 3 STATE GRID Brazil Holding S.A., Rio de Janeiro, Brazil

**Keywords:** Bird collision, Systematic review, Patent analysis, Deep learning, Biodiversity conservation

## Abstract

Bird collisions with transmission lines (TLs) cause significant bird mortality worldwide, yet technological solutions targeting this infrastructure remain critically underdeveloped. This systematic review synthesizes 58 peer-reviewed articles and 86 patents on bird detection and collision mitigation technologies for TL infrastructure, following the Preferred Reporting Items for Systematic Reviews and Meta-Analyses (PRISMA) 2020 guidelines. Although designed to capture the state of the art for TLs, most of the retrieved literature and patents originated from the aviation and wind energy sectors, where economic loss and human safety drive investment rather than biodiversity conservation. This asymmetry constitutes the central finding of the review: among studies directly targeting TL contexts, 73.7% focus exclusively on detection, and only 8.1% of patents are explicitly oriented toward biodiversity conservation outcomes. Among studies addressing detection, deep learning architectures dominate, particularly convolutional neural networks (CNNs); You Only Look Once (YOLO) variants are the most frequently reported, though they are not patentable as open architectures. Patent analysis, restricted to TLs and substations, revealed a sigmoidal growth trajectory, with artificial intelligence and computer vision (AI/CV) patents approaching saturation (*K* = 33.53) by 2023 and passive mechanical deterrence patents by 2024 (*K* = 44.33), driven predominantly by Chinese filings (95.3% of patents). However, a persistent structural gap remains: TL-specific applications represent a minority of the evidence base, and AI models trained predominantly on aviation and wind-farm datasets exhibit domain-shift limitations that constrain transferability to TL contexts. Addressing this gap requires investment in TL-specific datasets, field validation, and policy frameworks that position bird conservation as a primary driver of innovation.

**Supplementary Information:**

The online version contains supplementary material available at 10.1007/s10661-026-15647-w.

## Introduction

The expansion of energy distribution infrastructure, driven by the rapid global deployment of renewable energy, has raised significant concerns about its environmental impacts, particularly bird collisions with high-voltage power lines, hereinafter referred to as transmission lines (TLs) (Jenkins et al., 2011). Millions of birds die annually due to these hazardous interactions, posing a significant threat to vulnerable species and contributing to biodiversity loss (Bernardino et al., 2018; Jenkins et al., 2010, 2011). Numerous bird species are affected by TLs, particularly raptors and migratory birds, whose flight paths frequently overlap with areas where energy infrastructures are located, increasing their exposure to collision risk (Eccleston & Harness, 2018; Ferrer et al., 2020; Gauld et al., 2022).

Although the environmental impacts caused by TLs extend to other taxonomic groups, birds are disproportionately affected, mainly due to direct mortality from collisions with overhead wires (Biasotto & Kindel, 2018). Furthermore, TLs act as artificial barriers that disrupt bird movement between breeding, feeding, and migratory habitats, contributing significantly to the barrier effect and habitat fragmentation (Drewitt & Langston, 2006; C. Strevens et al., 2008; Richardson et al., 2017; Martins et al., 2023).

While the expansion of energy infrastructure is essential for the global energy transition, it significantly increases the likelihood of bird interactions with TLs and associated structures (Neri et al., 2019). To mitigate these impacts, various technologies have been developed and implemented globally (Bernardino et al., 2018, 2019; Garcia-Rosa, 2022). Among them, visual deterrents such as bird-friendly line markers and flappers are widely used to enhance line visibility, particularly under low-light conditions (Martin, 2022; Ngila et al., 2023). Studies suggest that these devices can reduce bird mortality by up to 70% compared to unmarked lines for susceptible species, although effectiveness remains species-dependent and was not demonstrated for all groups, such as great bustards (Ferrer et al., 2020; Shaw et al., 2021). However, this variability depends on species-specific traits, habitat characteristics, and local environmental conditions (Barrientos et al., 2011; Ferrer et al., 2020). No single mitigation measure is universally effective, often requiring customization based on the species at risk and the physical context of the infrastructure (Ferrer et al., 2020). Factors such as line height, surrounding vegetation, flight altitudes, and visibility conditions all influence performance (Travers, 2023).

These limitations highlight the need for more adaptive and responsive solutions, particularly those leveraging artificial intelligence. AI-based systems offer the potential for dynamic, site-specific, and species-specific detection and mitigation, addressing the rigidity and generalization constraints of conventional approaches (Bernardino et al., 2018; Reinhardt et al., 2025). Traditional monitoring methods, based on carcass searches and ground surveys, are labor-intensive, costly, temporally constrained, and prone to detection biases, particularly in remote or inaccessible areas (Liang et al., 2024).

Recent advances in artificial intelligence and computer vision (AI/CV), and remote sensing have enabled the development of automated systems capable of real-time bird detection and collision risk assessment (Chabot & Francis, 2016). These advances are primarily driven by interdisciplinary collaborations among computer scientists, ecologists, engineers, and industry stakeholders, particularly in the aviation and wind energy sectors (McClure et al., 2020). The integration of remote sensing devices with AI techniques has become a valuable tool in addressing these challenges, enhancing the effectiveness and efficiency of bird detection while ensuring robust, continuous data collection on collisions (Liu et al., 2020; Xiang et al., 2024). Detection technologies refer to systems capable of identifying, tracking, or sensing bird presence in real-time, such as optical sensors, radar, or AI-driven computer vision. Mitigation technologies encompass the measures taken to reduce collision risk (Faisal et al., 2025; Garcia-Rosa, 2022; Jiang et al., 2023). We distinguish between passive mitigation, which includes static devices like visual deterrents (e.g., bird-friendly line markers) (Barrientos et al., 2011), and active mitigation, which involves a responsive action (e.g., light or sound deterrents and turbine curtailment) (Tomé et al., 2017).

Complementing scientific literature with patent analysis is methodologically valuable in this context for two reasons. First, patents capture technological developments that precede or substitute peer-reviewed publication, particularly in commercial and industrial contexts where intellectual property protection is prioritized over academic dissemination (Gao et al., 2013; Griliches, 1990). Second, the temporal trajectory of patent accumulation, modeled through technological lifecycle frameworks, provides a market-facing indicator of innovation maturity independent of ecological validation or field deployment (Andersen, 1999; Phan & Daim, 2013). Together, scientific literature and patent analysis offer complementary evidence streams: the former characterizes performance, limitations, and ecological relevance, while the latter maps the commercial and innovation landscape.

Our study aims to compile and analyze advancements in scientific and technological innovations for bird detection systems, evaluating their role in mitigating the impacts of expansion of the energy sector on birds. Specifically, this study investigates whether current technological innovations, particularly AI-based bird detection systems, are advancing quickly enough to mitigate bird collisions in the context of the accelerating global energy transition, while simultaneously supporting biodiversity conservation goals. We conducted a systematic review of scientific literature and patent databases to map the key detection and mitigation technologies developed over the past decades. This analysis included an assessment of their operational environments and technological functionalities, along with a qualitative discussion of implementation challenges and limitations. Additionally, we applied statistical modeling to characterize the technological lifecycle of these solutions, evaluating whether their development trajectory aligns with the urgent demands of sustainable energy expansion and effective biodiversity conservation.

## Methods

This systematic review, which includes both scientific publications (studies) and patents, was conducted following the Preferred Reporting Items for Systematic reviews and Meta-Analyses (PRISMA) guidelines (Page et al., 2021). The literature and patent searches were performed in January 2026 using the Scopus and Web of Science (WoS) databases for scientific publications, and *the Derwent Innovations Index* (DWPI) and Lens.org platform for patent records. The search was restricted to records published between 2005 and 2025, ensuring a temporally consistent and comparable dataset across all sources.

### Systematic literature review

A comprehensive literature search was performed using the Scopus and WoS databases, querying the fields Title, Abstract, Keywords, and Topic. The search strategy combined primary keywords (“bird” OR “avian”) with complementary terms related to energy infrastructure, collision risks, and mitigation technologies.

The search was structured into three main thematic blocks. The first block targeted infrastructure-related terms, using the Boolean string: (“high*voltage*line*” OR “overhead line*” OR “power*line*” OR “distribution line*” OR “transmission line*” OR “overhead wire*” OR “conductor*” OR “shield wire*” OR “pylon*” OR “tower*” OR “transmission tower*” OR “energy grid”). The second block focused on bird-related and collision risk terms, employing combinations such as: (“bird*” OR “avian*” OR “collision*” OR “bird*strike*” OR “carcass” OR “bird fatality” OR “flight behavio*r” OR “collision risk” OR “flight diverter*” OR “line marker*” OR “wire marking” OR “deterrent*”). The third block was designed to identify studies incorporating real-time object detection technologies, using the Boolean (“artificial intelligence” OR “AI” OR “machine learning” OR “deep learning” OR “computer vision” OR “object detection” OR “image classification” OR “image segmentation” OR “convolutional neural network*” OR “transformer*” OR “vision transformer” OR “transfer learning” OR “domain adaptation” OR “domain shift” OR “edge computing” OR “embedded” OR “real-time”).

All searches were limited to peer-reviewed articles published in English within subject areas relevant to this review, including environmental sciences, ecology, engineering, and environmental technology. Conference papers, data papers, book chapters, and editorials were excluded.

### Patent search and analysis

The patent search was conducted using the global database *Derwent World Patents Index, DWPI* (Pires et al., 2020). A complementary search was carried out on Lens.org to capture patents not indexed in Derwent, particularly from emerging jurisdictions. The combined corpus was deduplicated by an application number. The same three-block keyword structure described above was adapted for patent title, abstract, and claims fields.

Patents were included if they met at least one of the following criteria: (i) devices or methods designed to detect bird collisions or to mitigate such collisions, including mechanical, acoustic, or visual deterrents; or (ii) technologies employing computer vision for bird detection, comprising components such as data processing units, communication modules, warning systems, or bird diversion mechanisms (Martin & Shaw, 2010).

Patents were classified by infrastructure type (transmission lines and towers, substations, wind farms, airports, and others); primary technology category (passive mechanical deterrence, AI and computer vision, physical sensors, radar and wave-based systems); functional focus (detection, mitigation, both); and conservation versus safety orientation. The quantitative analysis was restricted to patents targeting transmission lines and substations.

Additionally, a complementary market survey was conducted to identify commercial products currently used for bird collision detection and mitigation. This step was necessary because not all bird detection technologies are patented, often due to factors such as commercial confidentiality, proprietary restrictions, rapid technological obsolescence, or high costs associated with patenting (Gollin, 1991).

#### Technological growth modeling

To model the technological lifecycle of the patent corpus, multiple models were evaluated separately for each patent technology category. The Shapiro-Wilk test (Shapiro & Wilk, 1965) was applied to assess data normality prior to model selection. As the data did not follow a normal distribution, non-linear models were selected (Andersen, 1999; Gao et al., 2013), including a generalized additive model (GAM), an exponential model using non-linear least squares (NLS), and the Boltzmann sigmoidal model. These models are appropriate for capturing non-linear temporal growth patterns commonly associated with technological adoption curves describing the relationship between the predictor variable (year) and the response variable (cumulative number of patents) (Burnham & Anderson, 2002; Gao et al., 2013). All analyses were performed using R version 4.3.3 (R Core Team, 2024). The “mgcv” package was used to fit GAM models with spline-based smoothers (Wood, 2010). For the exponential model, we used the “nls” (non-linear least squares) function available in R base package (Bates & Watts, 1988). This model is suitable when the relationship between variables is best represented by an exponential function.

To evaluate the performance of the fitted models, we used *R*^2^ and adjusted *R*^2^, alongside the corrected Akaike information criterion (AICc) (Akaike, 1974; Burnham & Anderson, 2002) and included a null model as a reference for comparison. The null model represents a theoretical baseline assuming no relationship between time (year) and the cumulative number of patents, enabling us to assess whether the observed temporal patterns reflect meaningful technological growth rather than stochastic fluctuations. Model fit was evaluated by comparing AICc values, where a lower AICc relative to the null model indicates superior performance and supports the explanatory contribution of the predictors (Anderson, 2008; Burnham & Anderson, 2002; Burnham et al., 2011; Zuur et al., 2009).

## Results

### Systematic literature and patent review

**Fig. 1:**
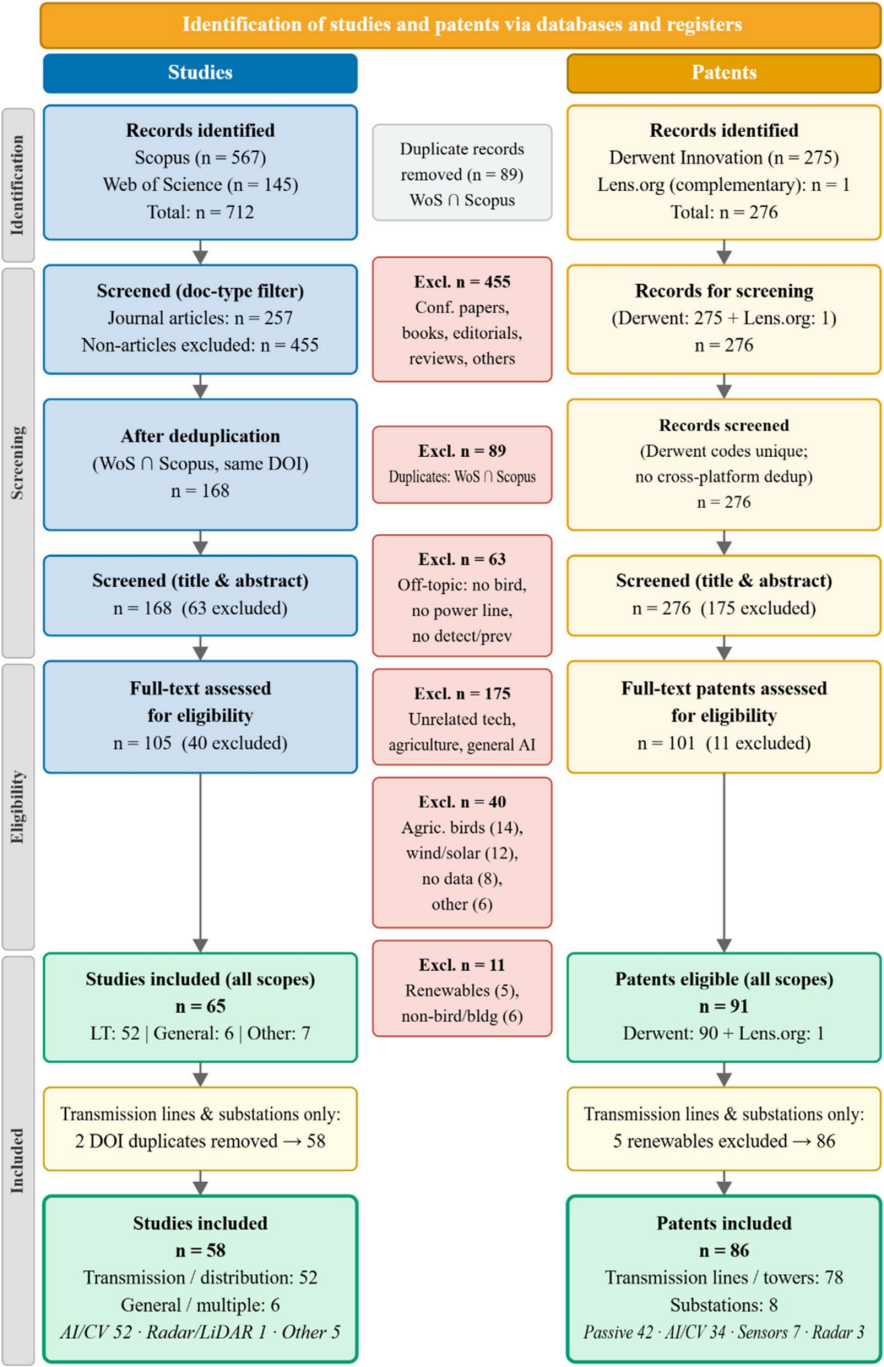
PRISMA 2020 flow diagram illustrating the identification, screening, eligibility, and inclusion stages for scientific publications (left) and patents (right). The workflow integrates records from bibliographic databases (Scopus and Web of Science) and patent databases (Derwent Innovation and Lens.org), with final inclusion restricted to transmission lines and substations for quantitative analysis

Following title and abstract screening, studies were assessed for full-text eligibility, resulting in 65 records meeting the inclusion criteria. After removing cross-platform duplicates and restricting the scope to transmission lines and substations, 58 studies were retained for quantitative analysis. In parallel, 86 patents specific to transmission lines and substations were selected from the 276 records identified (Fig. 1).

**Fig. 2:**
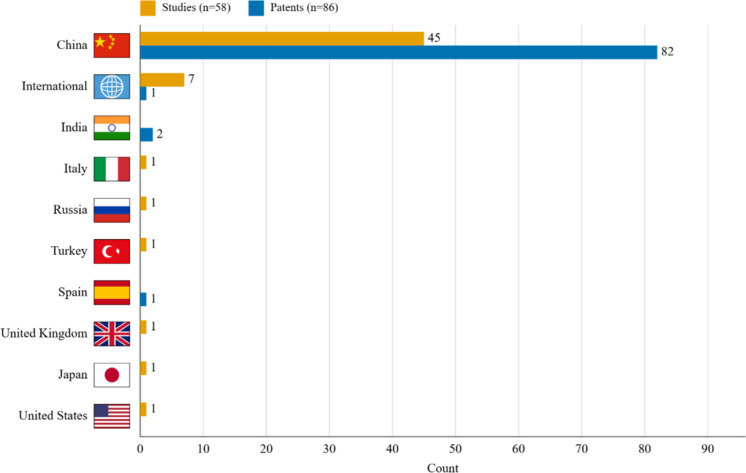
Geographic distribution of eligible studies (*n* = 58, yellow squares) and patents (*n* = 86, blue circles) by country of origin. The pronounced concentration of patents in China (*n* = 82; 95.3%) contrasts with the broader geographic spread of peer-reviewed studies, reflecting distinct national patenting incentive structures rather than genuine technological diversity

Although the search was designed to identify technologies relevant to transmission line (TL) contexts, studies addressing other infrastructure sectors were also retrieved, particularly airports (*n* = 6) and wind farms (*n* = 8). These were retained under the pre-defined transferability criterion. Among the included studies, 38 addressed TLs directly, while 6 considered general or multiple infrastructure contexts, resulting in a broader evidence base of 44 studies with direct or transferable relevance to TLs (Fig. 2). This structural imbalance constitutes one of the central outcomes of this review: most AI-based detection research and patent activity targets aviation and wind energy contexts, where economic loss and operational safety provide stronger incentives for investment than biodiversity conservation alone (Bernardino et al., 2018; D’Amico et al., 2019).

**Fig. 3:**
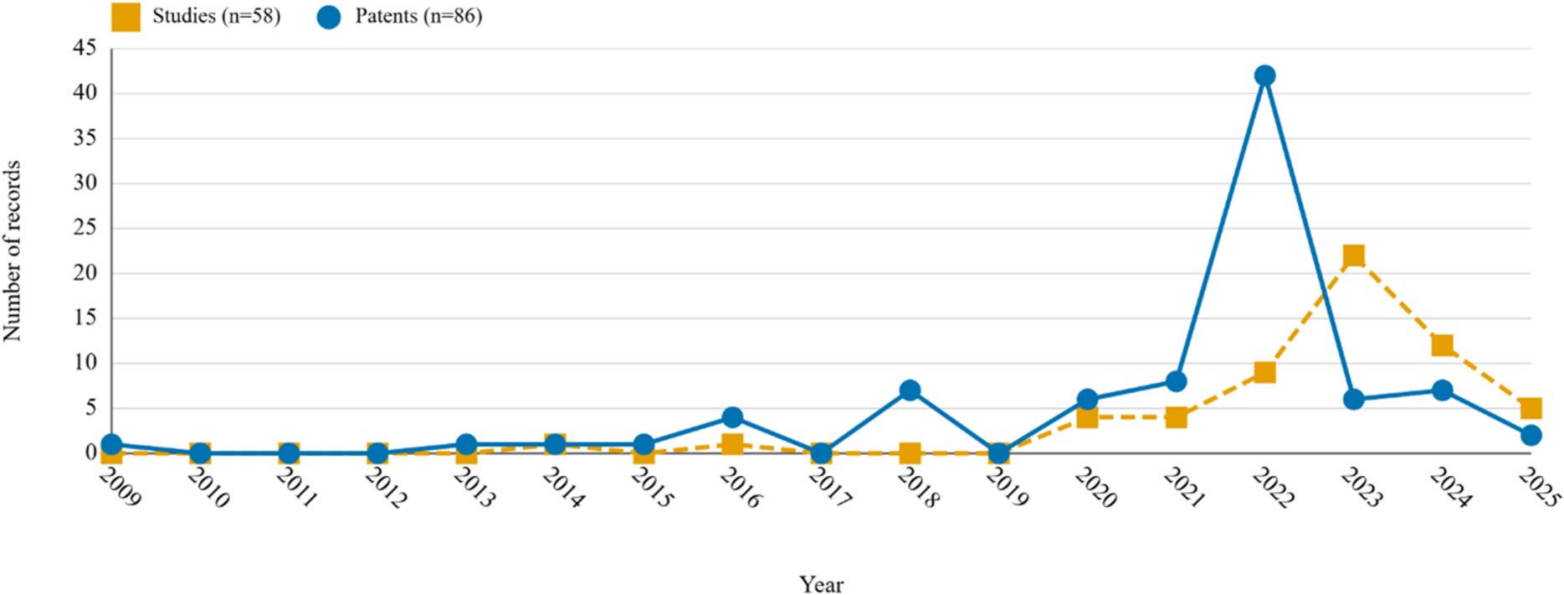
Annual number of eligible studies (*n* = 58, orange squares) and patents (*n* = 86, blue circles; transmission lines and substations) from 2009 to 2025

The temporal distribution of records indicates a marked intensification of both scientific and technological production after 2019, with a particularly pronounced surge in patenting activity in 2022, followed by a decline and stabilization in subsequent years (Fig. 3). While early activity (2009–2013) was negligible, the period from 2014 onwards shows a gradual increase, becoming sharply accentuated in the last 5 years. In contrast, scientific studies exhibit a more delayed but sustained growth pattern, peaking in 2023 and remaining relatively high thereafter. Overall, the dataset comprises 86 patents and 58 studies, reflecting a stronger recent expansion of technological outputs relative to academic production.

**Fig. 4:**
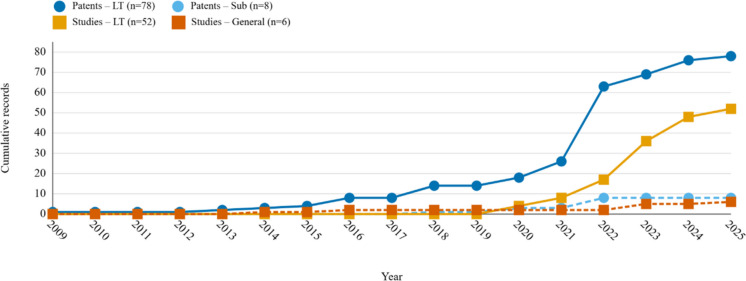
Cumulative growth of eligible records by infrastructure environment (2009–2025). Patent series: dark blue filled circles (transmission lines and towers, *n* = 78) and light blue filled circles connected by a dashed line (substations, *n* = 8). Study series: orange filled squares connected by a dashed line (transmission lines, *n* = 52) and dark orange filled squares connected by a dashed line (general/multiple environments, *n* = 6)

Regarding technological scope, among the 86 patents analyzed, 78 (90.7%) are associated with transmission lines and towers, whereas 8 (9.3%) target substations (Fig. 4). The cumulative curves highlight a clear acceleration in patent filings after 2021, especially for transmission line applications, suggesting a rapid scaling phase in technological development. Scientific studies follow a similar but less steep trajectory, with transmission line-focused research (*n* = 52) dominating over general studies (*n* = 6), indicating a strong alignment between research efforts and infrastructure-related risks to avifauna.

**Fig. 5:**
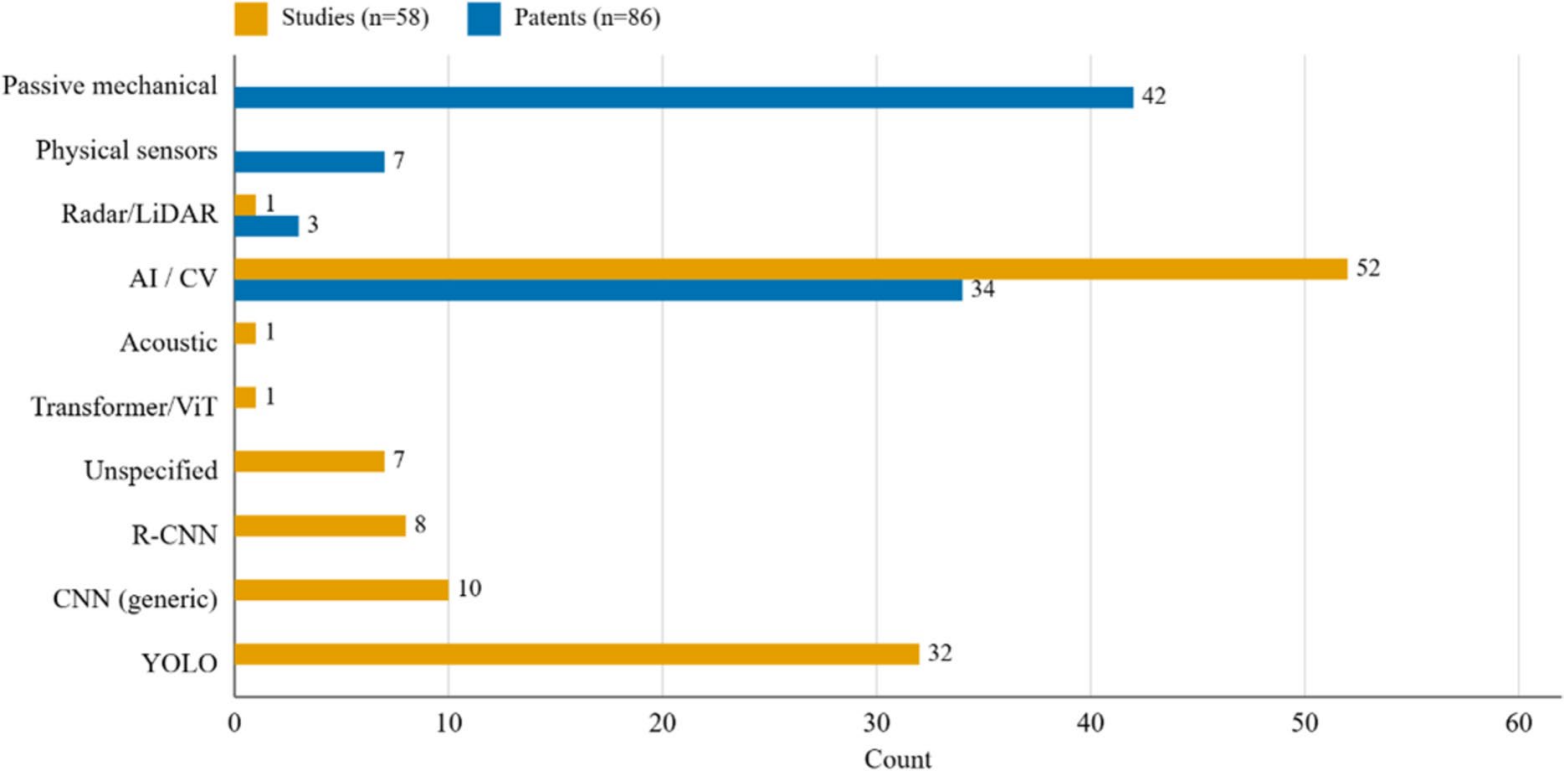
Distribution of technological approaches across eligible studies (*n* = 58, orange) and patents (*n* = 86, blue; restricted to transmission lines and substations)

**Fig. 6:**
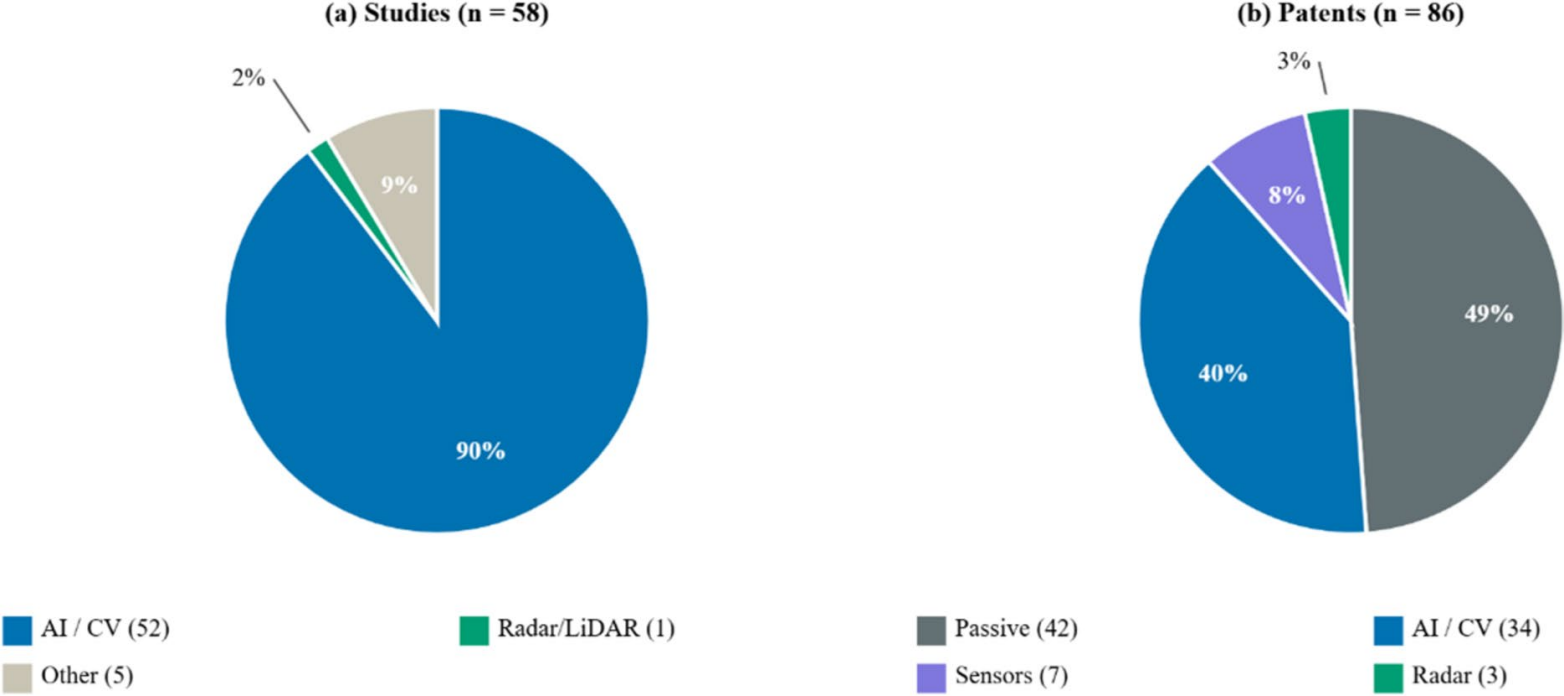
Proportional distribution of primary technological approaches in **a** eligible studies (*n* = 58) and **b** eligible patents (*n* = 86; restricted to transmission lines and substations)

From a technological perspective, the distribution of approaches reveals distinct patterns between patents and scientific studies (Fig. 5). Patent activity is dominated by passive mechanical systems (*n* = 42), reflecting a prevalence of mitigation-oriented solutions based on physical deterrence. In contrast, scientific studies are overwhelmingly concentrated in AI/CV approaches (*n* = 52), with a strong emphasis on deep learning architecture (Fig. 6). Within this group, convolutional neural networks (CNNs), particularly YOLO-based models (*n* = 32), are the most frequently applied techniques, followed by generic CNN implementations (*n* = 10) and region-based CNNs (R-CNN; *n* = 8). Other detection paradigms, including transformer-based models such as detection transformer (DETR) and variants, acoustic monitoring, and radar/LiDAR (light detection and ranging) systems, remain comparatively underrepresented.

Focusing on studies directly targeting transmission line (TL) contexts (*n* = 38), the distribution between detection and mitigation is markedly asymmetrical: 28 studies (73.7%) focus exclusively or primarily on detection, 6 (15.8%) on mitigation, and 4 (10.5%) integrate both approaches. Species-level identification is reported in only 7 studies (18.4%), whereas the majority rely on object-level detection, substantially limiting their ecological applicability for conservation planning.

**Fig. 7:**
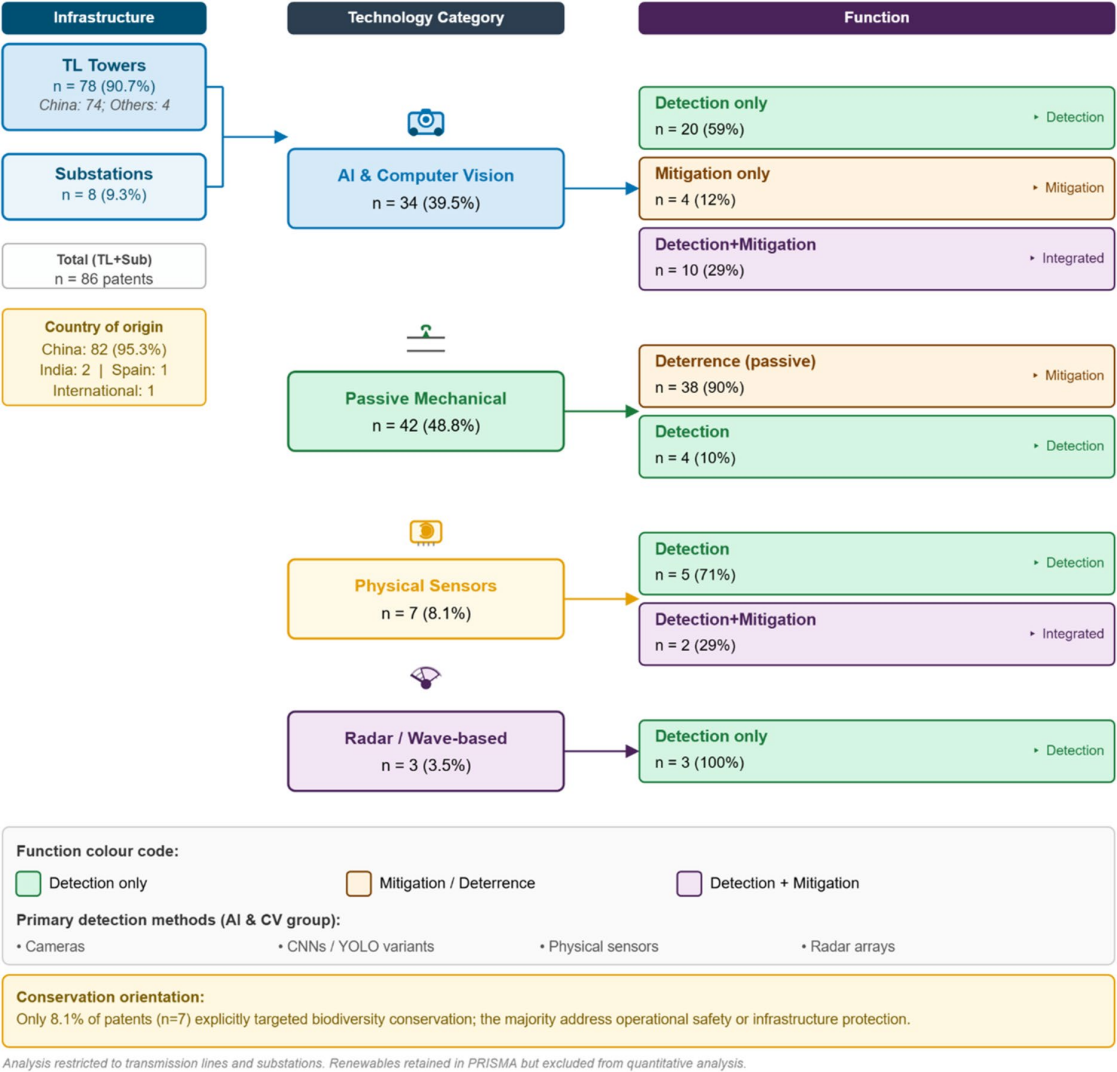
Classification of included patents (*n* = 86; restricted to transmission lines (TLs) and substations) by technology category and functional approach. Four technology categories are distinguished: artificial intelligence and computer vision (AI/CV; *n* = 34), passive mechanical systems (*n* = 42), physical sensors (*n* = 7), and radar/wave-based systems (*n* = 3). Functional classes include detection only, mitigation/deterrence, and integrated detection and mitigation, with category-specific distributions shown for each technology group

Among the 86 patents analyzed, 78 (90.7%) targeted transmission lines and towers, while 8 (9.3%) targeted substations. Classified by functional focus, 51 patents (59.3%) addressed detection, 22 (25.6%) addressed mitigation only, and 13 (15.1%) addressed integrated detection and mitigation systems (Fig. 7; Supplementary Material S1). Patents explicitly oriented toward biodiversity conservation outcomes were rare (*n* = 7; 8.1%), underscoring the predominantly safety-driven nature of innovation in this domain.

### Model selection and performance evaluation for predicting patent growth over time

Model selection indicated that the Boltzmann sigmoidal model was the most parsimonious in describing the relationship between year and cumulative patent counts in both technology groups analyzed. A total of four models were evaluated using the corrected Akaike information criterion (AICc), degrees of freedom (*df*), and model weights (*Wi*) (Table 1). Model selection was conducted independently for artificial intelligence and computer vision (AI/CV) patents (*n* = 34) and passive mechanical patents (*n* = 42), reflecting their distinct growth dynamics.

**Table 1:** Model selection results based on ΔAICc for models describing the relationship between year and cumulative patent counts, fitted separately for artificial intelligence and computer vision (AI/CV) patents (*n* = 34) and passive mechanical patents (*n* = 42)

Models	*R*^2^	*R*^2^ adjusted	ΔAICc	*df*	*Wi*
AI and computer vision (***n*** = 34)					
Boltzmann	0.969	0.969	**0.000**	4	**0.999**
Exponential (NLS)	0.944	0.944	11.820	2	0.001
GAM	0.971	0.963	28.440	9	< 0.001
Null	0.000	0.000	118.630	1	< 0.001
Passive mechanical (***n*** = 42)					
Boltzmann	0.981	0.981	**0.000**	4	**0.997**
Exponential (NLS)	0.961	0.961	10.540	2	0.002
GAM	0.983	0.977	27.110	9	< 0.001
Null	0.000	0.000	131.220	1	< 0.001

Note: *AICc*, corrected Akaike information criterion; *ΔAICc*, difference in *AICc* relative to the best-performing model within each group; *df*, degrees of freedom; *Wi*, Akaike weight. Model selection was conducted independently for each patent technology group; therefore, AICc values are not directly comparable across groups. For generalized additive models (GAMs), (*df*) represent the sum of the effective degrees of freedom (*edf*) associated with the smoothing terms, reflecting model flexibility rather than the number of independently estimated parameters. Bold values indicate the best-supported model within each technology group (*ΔAICc* = 0; highest Akaike weight, *Wi*)

For AI/CV patents, the Boltzmann model yielded ΔAICc = 0.000 and *Wi* = 0.999, indicating overwhelming support relative to competing models. The exponential model (NLS) yielded ΔAICc = 11.820 (*df* = 2; *Wi* = 0.001), indicating substantially poorer fit despite its lower complexity. The GAM (ΔAICc = 28.440; *df* = 9; *Wi* < 0.001) showed minimal support, reflecting an unfavorable trade-off between flexibility and parsimony. The null model (ΔAICc = 118.630; *df* = 1) exhibited negligible weight, confirming a strong temporal signal in cumulative AI/CV patent growth.

For passive mechanical patents, the Boltzmann model was similarly well supported (ΔAICc = 0.000; *Wi* = 0.997). The exponential model yielded ΔAICc = 10.540 (*df* = 2; *Wi* = 0.002), followed by the GAM (ΔAICc = 27.110; *df* = 9; *Wi* < 0.001) and the null model (ΔAICc = 131.220; *df* = 1; *Wi* < 0.001). These results confirm the Boltzmann model as the most appropriate for describing cumulative patent growth in both groups, providing the best balance between goodness-of-fit and model complexity.

To further assess model performance, parameter estimates were evaluated in relation to their ability to describe temporal accumulation patterns (Table 2). For AI/CV patents, the exponential model yielded one statistically significant parameter (*b* = 0.270, *t* = 8.055, *p* < 0.001), while parameter a was not significant (*a* = 0.519, *t* = 2.024, *p* = 0.061), indicating an overall accelerating growth pattern. For passive mechanical patents, both parameters were significant (*a* = 1.249, *t* = 2.735, *p* = 0.015; *b* = 0.230, *t* = 9.100, *p* < 0.001). The GAM smooth term for year was highly significant in both groups (AI/CV: χ^2^ ≈ 763, *p* < 0.001; passive mechanical: χ^2^ ≈ 995, *p* < 0.001), indicating strong non-linear temporal structure, albeit with reduced parsimony relative to the Boltzmann model.

**Table 2:** Parameter estimates for models describing cumulative patent growth over time, fitted separately for artificial intelligence and computer vision (AI/CV) patents (*n* = 34) and passive mechanical patents (*n* = 42). Significant parameters (*P* ≤ 0.05) are shown in bold. For the Boltzmann model, *K* (= A2) represents the asymptotic saturation value, A1 the initial state, *x₀* the inflection point (i.e., the year of maximum growth rate), and dx the transition breadth, representing the duration of the growth phase (in years). For generalized additive models (GAMs), *edf* denotes the effective degrees of freedom of the smooth term, Ref.*df* the reference degrees of freedom used for significance testing, and *Chi.sq* the approximate chi-squared statistic associated with the smooth term

Models	Coefficients	Estimate	Std. error	*t* value	Pr(>|*t*|)
AI and computer vision (***n*** = 34)					
Boltzmann	*A1*	**1.561**	0.298	5.238	**< 0.001**
	*K (A2)*	**33.528**	12.440	2.695	**0.018**
	*x0*	**2021.515**	0.412	> 999	**< 0.001**
	*dx*	**0.686**	0.218	3.147	**0.008**
Exponential (NLS)	*a*	0.519	0.256	2.024	0.061
	*b*	**0.270**	0.034	8.055	**< 0.001**
GAM	Intercept	2.241	2.269	0.988	0.340
	Year (smooth)	Edf ≈ 8	Ref.df ≈ 8	Chi.sq ≈ 763	**< 0.001**
Null	Intercept	**9.059**	3.030	2.990	**0.009**
Passive mechanical (***n*** = 42)					
Boltzmann	*A1*	**2.586**	0.421	6.143	**< 0.001**
	*K (A2)*	**44.327**	18.720	2.368	**0.034**
	*x0*	**2021.131**	0.387	> 999	**< 0.001**
	*dx*	**1.057**	0.264	4.004	**0.002**
Exponential (NLS)	*a*	**1.249**	0.457	2.735	**0.015**
	*b*	**0.230**	0.025	9.100	**< 0.001**
GAM	Intercept	3.093	2.513	1.231	0.239
	Year (smooth)	Edf ≈ 8	Ref.df ≈ 8	Chi.sq ≈ 995	**< 0.001**
Null	Intercept	**13.353**	3.802	3.512	**0.003**

**Fig. 8:**
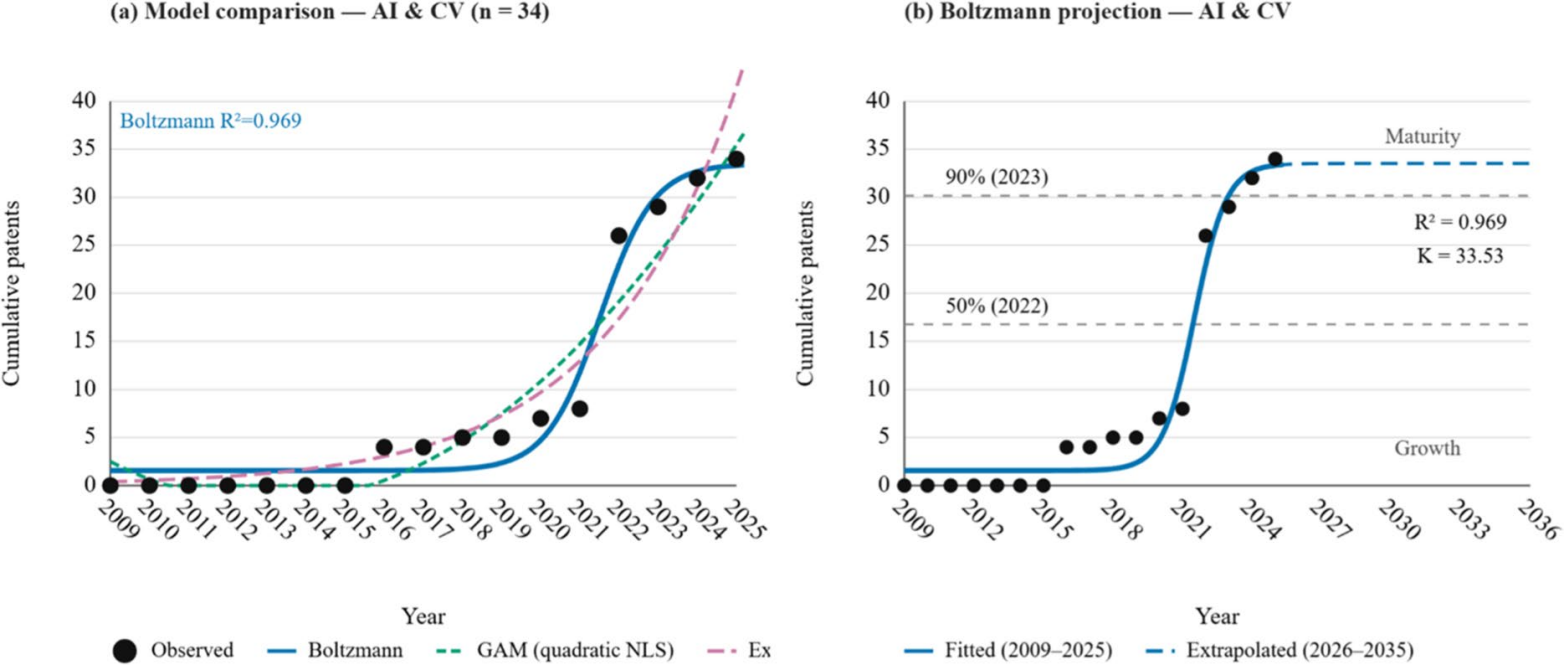
Boltzmann sigmoidal model selection and projection for artificial intelligence and computer vision (AI/CV) patents (*n* = 34). **a** Model comparison of cumulative patents: Boltzmann (blue solid; *Wi* = 0.999; *R*^2^ = 0.969), exponential model (green dashed), and generalized additive model (GAM; pink dashed). **b** Projection based on the Boltzmann fit, indicating an inflection point around 2021–2022 (*x*₀ = 2021.5) and approach to saturation (90% of *K* = 33.53) by approximately 2023. The narrow transition breadth (dx = 0.686) indicates a temporally concentrated growth phase

**Fig. 9:**
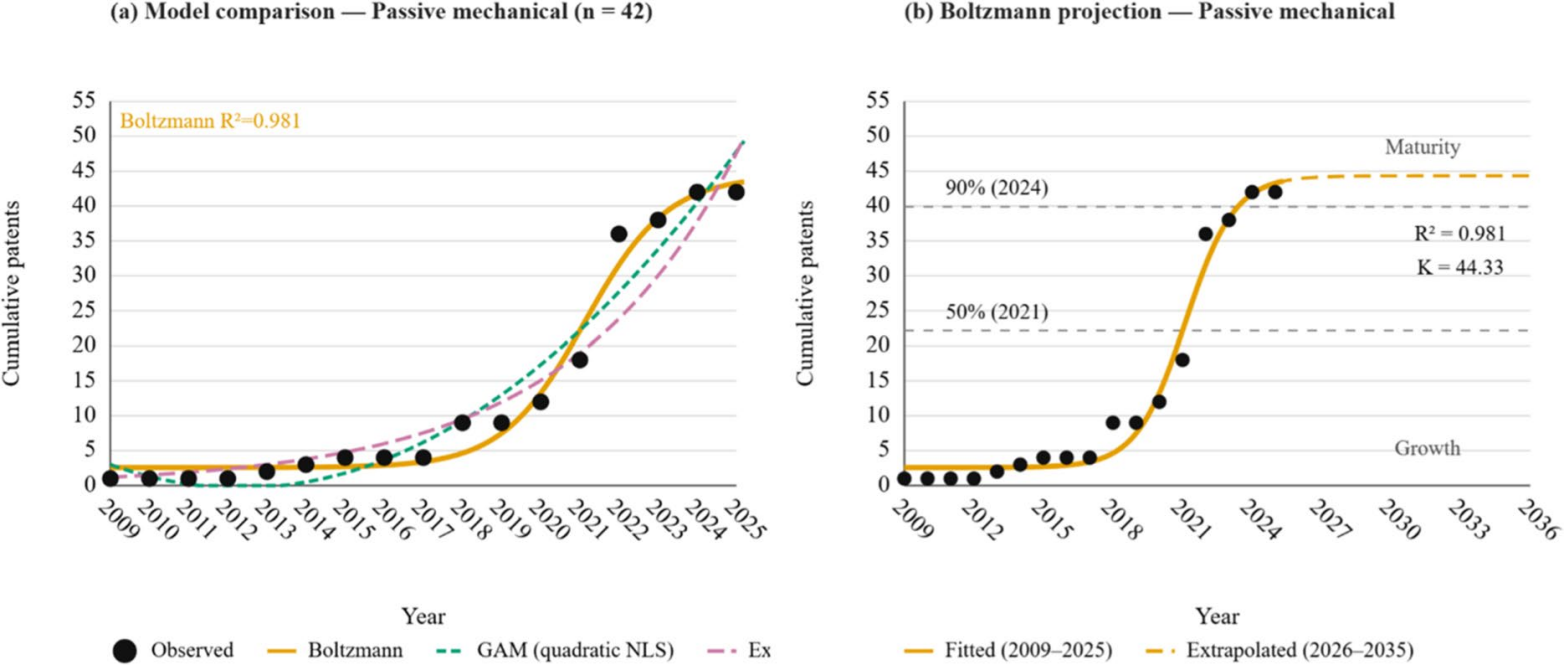
Boltzmann sigmoidal model selection and projection for passive mechanical patents (*n* = 42). **a** Model comparison: Boltzmann (amber solid; *Wi* = 0.997; *R*^2^ = 0.981), exponential model (green dashed), and generalized additive model (GAM; pink dashed). **b** Projection indicating an inflection point around 2021 (*X*₀ = 2021.1) and approach to saturation (90% of *K* = 44.33) by approximately 2024. The broader transition breadth (*dx* = 1.057), approximately 54% wider than that of the AI/CV group, indicates a more gradual growth phase

The Boltzmann model produced the most informative parameter estimates. For AI/CV patents, the estimated asymptotic saturation value was *K* = 33.528 (SE = 12.440, *t* = 2.695, *p* = 0.018), with initial state A1 = 1.561 (SE = 0.298, *t* = 5.238, *p* < 0.001), inflection point at *X*₀ = 2021.5 (SE = 0.412, *p* < 0.001), and transition breadth dx = 0.686 (SE = 0.218, *t* = 3.147, *p* = 0.008), indicating a narrow and temporally concentrated growth phase (Fig. 8). For passive mechanical patents, estimates were *K* = 44.327 (SE = 18.720, *t* = 2.368, *p* = 0.034), A1 = 2.586 (SE = 0.421, *t* = 6.143, *p* < 0.001), *X*₀ = 2021.1 (SE = 0.387, *p* < 0.001), and *dx* = 1.057 (SE = 0.264, *t* = 4.004, *p* = 0.002), indicating a broader transition phase approximately 54% wider than that observed for the AI/CV group (Fig. 9). In both groups, all Boltzmann parameters were statistically significant (*p* ≤ 0.05), with wider uncertainty associated with the asymptotic parameter *K*, reflecting the inherent limitations of estimating upper bounds from finite time series.

## Discussion

Our results reveal a pronounced structural asymmetry between scientific research and technological development in bird detection and bird collision mitigation systems. While the scientific literature is strongly dominated by artificial intelligence and computer vision (AI/CV) approaches (*n* = 52 of 58 studies), patent activity remains largely concentrated in passive mechanical mitigation systems, such as bird flight diverters (*n* = 42 of 86 patents; Figs. 5 and 6). This divergence reflects distinct innovation pathways, in which advances in detection technologies have not yet been fully translated into deployable mitigation solutions, particularly for bird conservation within TLs. The temporal pattern of this divergence is informative: patent activity surged sharply after 2019, reaching a peak in 2022 driven predominantly by Chinese infrastructure monitoring filings, before declining and stabilizing in subsequent years, while scientific publications show a more gradual but sustained growth trajectory, peaking in 2023 (Fig. 3). This asymmetry in timing and source suggests that the observed growth is largely disconnected from biodiversity conservation demand. Despite growing efforts, the overall representation of scientific studies (3.42%) and patents (1.42%) within the broader technological landscape remains relatively low, likely reflecting the inherent complexity of integrating bird monitoring requirements with scalable technological innovation (Fu et al., 2024).

Since 2020, there has been a sharp acceleration in both scientific publications and patent filings, particularly in computer vision technologies powered by AI. These advances have significantly improved bird detection accuracy compared to earlier detection methods such as optical, thermal, acoustic sensors, and radar systems (Chen et al., 2024; Elef et al., 2025; Fu et al., 2024; Kondaveeti et al., 2025). A rapid expansion of solutions based on deep learning models, especially convolutional neural networks (CNNs) and real-time object detection architectures, is evident. Within the AI/CV group, YOLO-based variants (*n* = 32) are the most frequently applied techniques, followed by generic CNN implementations (*n* = 10) and region-based CNNs (R-CNN; *n* = 8). Recent studies directly targeting TL contexts report mean detection accuracies ranging from 91 to 95%, including YOLO-based systems achieving 92.2% in foreign-object detection on transmission towers (Jiang et al., 2023), 94.2% in species detection on TL infrastructure (Xue et al., 2025), and integrated detection-repellence systems reaching 91.21% accuracy (Wu et al., 2023). Species classification in TL contexts has also demonstrated over 94% accuracy using lightweight deep learning models (Qiu et al., 2024). These technologies have demonstrated high accuracy and strong potential for field deployment (Alqaysi et al., 2021; Alzadjali et al., 2024), enabling effective bird detection across sectors such as aviation and wind energy (Alzadjali et al., 2024; Bošnjaković et al., 2025; Gémard et al., 2025). They contribute not only to human safety and the continuity of energy generation and distribution but also, when properly implemented, to biodiversity conservation (Alzadjali et al., 2024; Faisal et al., 2025; Sun et al., 2023). Nevertheless, their translation into effective mitigation strategies for bird conservation within TLs remains comparatively limited.

The post-2019 acceleration is best interpreted as the result of converging structural drivers rather than a single causal mechanism. First, the proliferation of pre-trained deep learning architectures substantially reduced the technical barrier for detection model development, enabling rapid adoption across research groups without specialist computer vision expertise (Alzadjali et al., 2024; Bošnjaković et al., 2025). Second, the expansion of offshore wind energy infrastructure in Europe and Asia, driven by regulatory mandates and net-zero commitments, created strong commercial incentives for bird detection investment, generating methodological and technological spillover into adjacent infrastructure contexts (Gémard et al., 2025; Principato et al., 2023). Third, the strategic prioritization of AI-enabled infrastructure monitoring in China produced a highly concentrated surge in patent activity in 2022 (Fig. 2), reflected in the narrow transition breadth observed for AI/CV technologies (*dx* = 0.686) relative to passive mechanical deterrence (*dx* = 1.057; Figs. 8 and 9). With 82 of 86 patents (95.3%) originating from China, this concentration reflects national infrastructure monitoring policy priorities rather than a genuine diversification of conservation-oriented innovation globally (D’Amico et al., 2019). Notably, these structural drivers are largely external to biodiversity conservation priorities, which helps explain the persistent underrepresentation of TL-specific and conservation-oriented innovation: only 7 of 86 patents (8.1%) are explicitly oriented toward biodiversity outcomes (Fig. 7) (Bernardino et al., 2018; Ngila et al., 2023).

This imbalance is further reinforced by the functional distribution of approaches across the reviewed evidence. Focusing on studies directly targeting TL contexts (*n* = 38), 28 (73.7%) focus exclusively or primarily on detection, 6 (15.8%) on mitigation, and only 4 (10.5%) integrate both approaches (Fig. 5). Moreover, species-level identification is reported in only 7 studies (18.4%), whereas the majority rely on object-level detection (Li et al., 2025; Qiu et al., 2024), substantially limiting ecological applicability for conservation planning, given that collision risk is strongly mediated by species-specific traits such as morphology, flight behavior, and sensory ecology. This functional asymmetry likely reflects a broader structural gap: publishing detection benchmarks requires relatively standardized laboratory or controlled-environment datasets, whereas demonstrating mitigation effectiveness requires long-term field campaigns, infrastructure integration, and population-level outcome metrics, none of which is readily available for TL contexts. The few studies combining detection and active deterrence in TL settings demonstrate technical feasibility (Wu et al., 2023), but their limited number underscores the translational gap between detection capability and validated conservation outcomes, a gap that this review identifies as one of the most critical priorities for future research (Tuia et al., 2022). A further dimension of this functional asymmetry warrants attention. A substantial body of literature within the reviewed corpus addresses the detection of bird nests on transmission towers and conductors, a distinct operational problem driven by the fact that nests on energized equipment cause short-circuits and power outages. Recent studies report detection accuracies above 89% for nest identification on TL infrastructure using lightweight deep learning architecture specifically trained on tower inspection imagery (Meng et al., 2024; Xiang et al., 2024; Xu et al., 2025). Several patents in the corpus similarly target nest prevention and removal, employing physical barriers, robotic removal arms, and autonomous drone systems (e.g., CN208211343; CN110692619). This body of work demonstrates that TL-specific AI datasets and high-performance detection models are technically achievable. However, nest detection serves exclusively operational objectives: protecting energy infrastructure from damage caused by nesting activity. It does not address collision risk, species-level identification, or conservation planning. This contrast reinforces the structural argument above: the technical capacity to develop accurately, TL-specific AI models exists and has been demonstrated, but it has been directed toward operational reliability rather than biodiversity outcomes (Tuia et al., 2022).

Cumulative patent trajectories for both AI/CV and passive mechanical systems follow a sigmoidal pattern consistent with a technological S-curve (Figs. 8 and 9). Within the observed time series and under the assumptions of the fitted models, both groups appear to be approaching saturation, with AI/CV patents reaching approximately 90% of the asymptotic parameter (*K* = 33.53) by 2023, and passive mechanical systems by 2024 (*K* = 44.33). This apparent saturation should be interpreted cautiously, as it reflects the current structure of patent activity rather than definitive technological maturity or widespread deployment within TLs. AI/CV technologies exhibit a temporally concentrated growth phase (dx = 0.686), whereas passive mechanical systems show a broader and more gradual diffusion pattern (dx = 1.057), approximately 54% wider, likely reflecting differences in innovation cycles and commercial diffusion rates. Rather than indicating stagnation, this pattern is consistent with progression across technology readiness levels (TRLs), from exploratory development toward system validation and early-stage commercial deployment (Mankins, 2009). Importantly, the saturation of patent activity does not imply that the conservation challenge has been addressed: the body of work from which these trajectories derive is predominantly driven by aviation safety and wind-farm operational objectives, and TL-specific, ecologically validated applications remain conspicuously absent from the patent landscape (Fu et al., 2024).

Meeting the global targets for renewable energy expansion requires integrated environmental impact mitigation strategies in which autonomous bird monitoring systems emerge as strategic components (Alzadjali et al., 2024; Garcia-Rosa, 2022; Labianca et al., 2025). As wind and solar energy expand (IRENA, 2017; IEA, 2021; United Nations Framework Convention on Climate Change, 2023; International Renewable Energy Agency & Global Renewables Alliance, 2024), there is a growing need to modernize and extend TLs across ecologically sensitive landscapes, increasing cumulative collision and electrocution risks for avifauna (Bernardino et al., 2018; Biasotto & Kindel, 2018; Principato et al., 2023). Deploying combined monitoring strategies that integrate autonomous AI-powered systems with conventional methods is therefore a strategic approach for real-time collision and electrocution risk mitigation along TLs (Jiang et al., 2023; Wu et al., 2023). Continuous monitoring using sensors, cameras, and AI can significantly reduce collision risks and bird mortality, enabling the development of environmentally responsible energy infrastructure (Jiang et al., 2023; Principato et al., 2023). Their effectiveness, however, remains dependent on context-specific field validation and integration with existing mitigation frameworks.

These technological growth trajectories align with market projections that indicate a growing economic relevance of the sector. The bird detection and mitigation market is currently valued at approximately USD 132.90 million, with projections indicating it will reach USD 220.62 million by 2030, representing compound annual growth rates between 8.6 and 11.2% (Business Research Insights, 2024; Global Growth Insights, 2024; Stratview Research, 2024). However, a notably lower estimate of 5% has also been reported (Future Market Insights, 2024), reflecting differences in methodological approaches, market segmentation criteria, and assumptions regarding technological adoption rates. Such variability highlights the inherent uncertainty in forecasting emerging technology markets, which should be considered when interpreting these projections. Beyond market dynamics, broader analyses of AI applications in conservation reinforce the urgency of aligning technological development with ecological priorities (Labianca et al., 2025; Reynolds et al., 2025). This combination of growth trajectories and market forecasts reinforces the need to strengthen public policies, increase investment in applied research, and incorporate these technologies into regulatory frameworks for environmental licensing and energy infrastructure operation (Benner & Tushman, 2003; Griliches, 1990).

Nevertheless, a substantial gap persists regarding the direct application of these technologies to bird conservation within TLs (Biasotto et al., 2022; Reinhardt et al., 2025). A central limitation of the current evidence base is the implicit and largely unexamined assumption that technologies developed for aviation and wind energy sectors are directly transferable to TL contexts. Detection systems for airports operate in spatially bounded environments with stable infrastructure, fixed sensor platforms, and relatively predictable species assemblages (DeVault et al., 2013; Fu et al., 2024). Wind farms similarly benefit from defined rotor perimeters, continuous power supply, and commercially motivated monitoring. Transmission lines, by contrast, extend across hundreds to thousands of kilometers, traverse heterogeneous habitats and climatic zones, and lack permanent infrastructure for sensor deployment. Furthermore, the species assemblages at risk differ substantially, comprising not only large-bodied species such as great bustards, storks, pelicans, and cranes whose morphology and flight behavior directly influence collision risk (Gauld et al., 2022; Martin & Shaw, 2010), but also small migratory passerines during nocturnal passage, which present fundamentally distinct detection challenges. These structural differences indicate that technological readiness achieved in aviation and wind energy does not necessarily translate to TL applications (Gauld et al., 2022; Ngila et al., 2023).

Moreover, adverse weather conditions including fog, rain, and low-light environments significantly impair the performance of computer vision–based optical detection systems, as well as physical mitigation devices such as bird flight diverters, whose effectiveness is highly variable depending on species and context (Ferrer et al., 2020; Faisal et al., 2025). Although recent deep learning architectures such as YOLOv8 and YOLOv10 have achieved significant improvements, detecting small or distant birds remains one of the most challenging tasks for AI-based systems (Chalmers et al., 2025). There is still a strong geographic concentration of technological development, predominantly in China, the USA, and Europe, with limited validation and deployment in tropical regions and the Global South, which are home to some of the world’s greatest avian biodiversity (Biasotto & Kindel, 2018; Jenkins et al., 2010). AI models trained primarily on datasets from these regions are likely to underperform when applied to megadiverse tropical contexts, where morphological variability, vegetation complexity, and environmental conditions challenge model generalization (Baudchon et al., 2025; Tuia et al., 2022). Furthermore, hardware validated in temperate climates may fail when deployed under high humidity, heat, and seasonal precipitation conditions common to the Global South. This results in a significant conservation paradox, in which the most advanced mitigation tools are least accessible and least validated in the areas where biodiversity needs are greatest (Devarapalli, 2025).

A critical challenge remaining is the underutilization of the ecological data generated by these monitoring systems. In most cases, monitoring outputs are used primarily for operational purposes, while their potential to inform ecological research, population monitoring, and conservation planning remains largely unexplored (DeVault et al., 2013; Jenkins et al., 2010). Addressing this gap will require not only continued technological development but also cross-sector collaboration, standardized data protocols, and policy integration to enable scalable and effective solutions in the regions where they are most urgently needed (Tuia et al., 2022). Ultimately, the central finding of this review is not the absence of technology, but the misalignment between where innovation is occurring and where biodiversity conservation requires it most. Bridging this gap requires a deliberate shift in research, investment, and regulatory priorities toward TL-specific, ecologically validated applications, particularly in megadiverse regions of the Global South.

### Limitations

Despite its comprehensive scope, this study has several limitations. The systematic search was performed in January 2026 and therefore does not capture developments published after that date, which is particularly relevant given the rapid pace of AI-based detection research. The inclusion of studies from aviation, wind energy, and other infrastructure sectors, although justified by the pre-defined transferability criterion, introduces contextual heterogeneity that may affect the comparability of findings across application domains. Additionally, database coverage limitations and restricted access to regional patents and gray literature may result in a partial representation of the technological landscape, particularly for non-English and regionally filed patents (Booth et al., 2016). Patent descriptions are often intentionally broad or legally generic, limiting precise interpretation of functional scope, deployment status, and technological maturity (Europa Publications, 2022; Griliches, 1990; OECD Patent Statistics Manual, 2009). The reliance on titles and abstracts for initial eligibility screening may constrain the depth of information extracted from individual records; aspects such as operational field effectiveness, ecological impact, and deployment cost are not captured by this approach (Bernardino et al., 2018). Finally, the quantitative analyses were restricted to patents targeting transmission lines and substations, and findings therefore do not represent the broader patent landscape that includes wind farms and airport applications.

## Conclusions

This systematic review demonstrates that AI/CV-based technologies for bird detection and collision mitigation in TL infrastructure have advanced rapidly over the past decade, with sigmoidal patent growth trajectories indicating that both AI/CV and passive mechanical systems are approaching saturation. However, this technological momentum has not translated into equivalent progress for bird conservation within TLs. Most studies and patents originate from aviation and wind energy sectors, driven by economic and operational safety incentives rather than conservation mandates, and are geographically concentrated in China, with limited validation in the megadiverse tropical regions where avian biodiversity and collision risk are greatest.

The persistent gap in TL-specific, biodiversity-oriented applications represents the central finding of this review. Current evidence shows that detection technologies are available but largely untested under TL field conditions, that integrated detection-mitigation pipelines remain rare, and that species-level identification is largely absent from the TL literature. Addressing these gaps will require investment in TL-specific datasets and field validation programs, policy frameworks that explicitly position bird conservation as a primary driver of innovation rather than a secondary safety benefit, and stronger integration between ecological science and engineering development. The saturation of the current patent cycle signals a critical window: as the field transitions from active invention toward deployment and consolidation, conservation-specific requirements must be embedded into product development roadmaps to ensure that advancing AI/CV capabilities are directed toward the contexts where they are most urgently needed.

## Supplementary Information

Below is the link to the electronic supplementary material.

ESM 1 (DOCX 33.2 KB)

## Data Availability

All data generated or analyzed during this study are included in this published article and its supplementary information files.

## Declarations

All authors have read, understood, and complied as applicable with the statement on “Ethical responsibilities of Authors” as found in the Instructions for Authors.
