# Supplementary material for: Monitoring birds interacting with power lines: a systematic review of detection technologies and the persistent gap in transmission line applications
Source: Environ Monit Assess. 2026 Jul 25;198(8):879. doi: 10.1007/s10661-026-15647-w (PMC13401544; doi:10.1007/s10661-026-15647-w)
Supplement: Supplementary file 1 — (DOCX 33.2 KB) [file 10661_2026_15647_MOESM1_ESM.docx]

**Supplementary Material**

*Menezes et al. Environmental Monitoring and Assessment*

*Systematic review search performed in January 2026 Transmission Lines and Substations*

# Table S1. Patents included in the analysis (transmission lines and substations; n = 86)

**Note:** *Patents are classified by infrastructure type, detection method, mitigation approach, functional focus (Detection only / Mitigation only / Detection + Mitigation), and conservation orientation. Category codes:* ***Bird*** *= explicitly bird-focused;* ***Dual*** *= operational safety + conservation;* ***General*** *= safety/operational only. All patents represent Scope A records (TLs and substations). For the complete dataset including wind farms and airport patents, see the supplementary database files.*

| # | Patent Number | Year | Country | Infrastructure | Detection Method | Mitigation Method | Functional Focus | Category |
| --- | --- | --- | --- | --- | --- | --- | --- | --- |
| 1 | CN113951244 | 2022 | China | TL/Towers | AI/CV | Intelligent Systems | Detection + Mitigation | Bird |
| 2 | CN116369303 | 2023 | China | TL/Towers | Passive | Visual (Laser) | Mitigation | Bird |
| 3 | CN217928154 | 2022 | China | TL/Towers | Passive | Physical Barriers | Mitigation | Bird |
| 4 | CN114284970 | 2022 | China | TL/Towers | AI/CV | Acoustic | Detection + Mitigation | Bird |
| 5 | CN222140861 | 2024 | China | TL/Towers | AI/CV | Physical Barriers | Detection + Mitigation | Bird |
| 6 | CN214546808 | 2021 | China | TL/Towers | Passive | Physical Barriers | Mitigation | Bird |
| 7 | CN200965994 | 2009 | China | TL/Towers | Passive | Physical Barriers | Mitigation | Bird |
| 8 | CN216147105 | 2022 | China | TL/Towers | Physical Sensors | Physical Barriers | Detection | Dual |
| 9 | CN111241905 | 2020 | China | TL/Towers | AI/CV | Physical Barriers | Detection + Mitigation | Bird |
| 10 | CN113080185 | 2022 | China | TL/Towers | Radar/Wave | Physical Barriers | Detection + Mitigation | Bird |
| 11 | CN111972390 | 2020 | China | Substation | Passive | Visual (Laser) | Mitigation | Bird |
| 12 | CN218073238 | 2022 | China | Substation | Passive | Physical Barriers | Mitigation | Bird |
| 13 | CN120377115 | 2022 | China | TL/Towers | Passive | Acoustic | Mitigation | Bird |
| 14 | CN208581774 | 2018 | China | TL/Towers | Passive | Visual/Sound | Mitigation | Bird |
| 15 | CN221044127 | 2024 | China | TL/Towers | Passive | Physical Barriers | Mitigation | Bird |
| 16 | ES2526099 | 2015 | Spain | TL/Towers | Passive | Visual | Mitigation | Bird |
| 17 | CN107980762 | 2018 | China | TL/Towers | Passive | Intelligent Systems | Mitigation | Bird |
| 18 | CN116686820 | 2023 | China | TL/Towers | Radar/Wave | Physical Barriers | Detection | Dual |
| 19 | CN116686821 | 2023 | China | TL/Towers | AI/CV | Intelligent Systems | Detection + Mitigation | Dual |
| 20 | CN215380945 | 2021 | China | TL/Towers | Passive | Visual | Mitigation | Bird |
| 21 | CN216627265 | 2022 | China | TL/Towers | Passive | Physical Barriers | Mitigation | General |
| 22 | CN212237180 | 2021 | China | TL/Towers | Passive | Physical Barriers | Mitigation | Bird |
| 23 | CN223349460 | 2022 | China | TL/Towers | AI/CV | Physical Barriers | Detection + Mitigation | Dual |
| 24 | CN117356548 | 2022 | China | TL/Towers | Physical Sensors | Physical Barriers | Detection | Dual |
| 25 | CN208211343 | 2018 | China | TL/Towers | Passive | Visual | Mitigation | Bird |
| 26 | CN116773967 | 2023 | China | TL/Towers | Passive | Visual | Mitigation | Dual |
| 27 | CN112155004 | 2021 | China | TL/Towers | Passive | Acoustic | Mitigation | Bird |
| 28 | CN222869726 | 2024 | China | TL/Towers | AI/CV | Physical Barriers | Detection + Mitigation | Dual |
| 29 | CN110692619 | 2022 | China | TL/Towers | Passive | Intelligent Systems | Mitigation | General |
| 30 | CN209089786 | 2022 | China | TL/Towers | Passive | Physical Barriers | Mitigation | General |
| 31 | CN206165611 | 2022 | China | TL/Towers | AI/CV | Physical Barriers | Detection | Dual |
| 32 | CN111955448 | 2020 | China | TL/Towers | AI/CV | Visual | Detection + Mitigation | Bird |
| 33 | CN106259291 | 2018 | China | TL/Towers | Physical Sensors | Physical Barriers | Detection | Dual |
| 34 | CN201360129 | 2022 | China | TL/Towers | Passive | Acoustic | Mitigation | Bird |
| 35 | CN115997750 | 2022 | China | TL/Towers | Passive | Physical Barriers | Mitigation | General |
| 36 | CN222853021 | 2024 | China | TL/Towers | Passive | Physical Barriers | Mitigation | General |
| 37 | CN222905873 | 2024 | China | Substation | Physical Sensors | Physical Barriers | Detection | Dual |
| 38 | CN113359211 | 2022 | China | TL/Towers | AI/CV | Physical Barriers | Detection + Mitigation | Bird |
| 39 | CN120430129 | 2022 | China | TL/Towers | AI/CV | Physical Barriers | Detection + Mitigation | Dual |
| 40 | CN113678814 | 2022 | China | TL/Towers | AI/CV | Physical Barriers | Detection | Dual |
| 41 | CN120570267 | 2022 | China | TL/Towers | Passive | Physical Barriers | Mitigation | General |
| 42 | CN204132085 | 2022 | China | TL/Towers | AI/CV | Visual | Detection + Mitigation | Bird |
| 43 | CN112998004 | 2021 | China | TL/Towers | Passive | Physical Barriers | Mitigation | Bird |
| 44 | CN112889802 | 2021 | China | TL/Towers | Physical Sensors | Intelligent Systems | Detection | Dual |
| 45 | CN108651436 | 2018 | China | TL/Towers | Passive | Physical Barriers | Mitigation | General |
| 46 | CN117981744 | 2022 | China | Substation | Passive | Physical Barriers | Mitigation | General |
| 47 | CN115669646 | 2022 | China | TL/Towers | Passive | Physical Barriers | Mitigation | General |
| 48 | CN113016768 | 2022 | China | Substation | Physical Sensors | Physical Barriers | Detection | Dual |
| 49 | CN113498772 | 2022 | China | TL/Towers | Passive | Physical Barriers | Mitigation | General |
| 50 | CN105178679 | 2022 | China | TL/Towers | Passive | Physical Barriers | Mitigation | General |
| 51 | CN202958586 | 2022 | China | TL/Towers | Passive | Physical Barriers | Mitigation | General |
| 52 | CN113569981 | 2022 | China | TL/Towers | AI/CV | Physical Barriers | Detection | Dual |
| 53 | CN120448871 | 2022 | China | TL/Towers | AI/CV | Physical Barriers | Detection | Dual |
| 54 | CN120836530 | 2022 | China | TL/Towers | Passive | Physical Barriers | Mitigation | General |
| 55 | CN204167020 | 2022 | China | TL/Towers | Passive | Visual | Mitigation | Bird |
| 56 | IN202441070720 | 2024 | India | TL/Towers | AI/CV | Physical Barriers | Detection + Mitigation | Dual |
| 57 | CN220756315 | 2022 | China | Substation | Passive | Physical Barriers | Mitigation | General |
| 58 | CN107549161 | 2018 | China | TL/Towers | Passive | Physical Barriers | Mitigation | General |
| 59 | CN215075017 | 2021 | China | TL/Towers | Passive | Physical Barriers | Mitigation | Dual |
| 60 | CN217217963 | 2022 | China | TL/Towers | Passive | Physical Barriers | Mitigation | Dual |
| 61 | CN211581358 | 2020 | China | TL/Towers | Passive | Physical Barriers | Mitigation | Dual |
| 62 | CN217446383 | 2022 | China | TL/Towers | AI/CV | Physical Barriers | Detection + Mitigation | Dual |
| 63 | CN211793987 | 2020 | China | TL/Towers | Passive | Physical Barriers | Mitigation | Dual |
| 64 | CN205884521 | 2022 | China | TL/Towers | Passive | Physical Barriers | Mitigation | Dual |
| 65 | CN108055198 | 2018 | China | TL/Towers | Passive | Physical Barriers | Mitigation | Dual |
| 66 | CN203302232 | 2022 | China | Substation | Passive | Physical Barriers | Mitigation | Dual |
| 67 | CN112291001 | 2021 | China | TL/Towers | Passive | Physical Barriers | Mitigation | General |
| 68 | CN113261478 | 2021 | China | TL/Towers | Passive | Acoustic | Mitigation | Bird |
| 69 | CN213279691 | 2021 | China | TL/Towers | Passive | Physical Barriers | Mitigation | General |
| 70 | CN215600083 | 2022 | China | TL/Towers | Passive | Visual | Mitigation | Bird |
| 71 | CN115281079 | 2022 | China | TL/Towers | Passive | Acoustic | Mitigation | Bird |
| 72 | CN217469106 | 2022 | China | TL/Towers | Passive | Physical Barriers | Mitigation | General |
| 73 | CN218789495 | 2022 | China | TL/Towers | Passive | Physical Barriers | Mitigation | General |
| 74 | CN219020008 | 2022 | China | Substation | Passive | Physical Barriers | Mitigation | General |
| 75 | CN219124173 | 2022 | China | TL/Towers | Passive | Physical Barriers | Mitigation | General |
| 76 | CN219146394 | 2022 | China | TL/Towers | Passive | Physical Barriers | Mitigation | General |
| 77 | CN219182427 | 2022 | China | TL/Towers | Passive | Physical Barriers | Mitigation | General |
| 78 | CN219843823 | 2022 | China | TL/Towers | Physical Sensors | Physical Barriers | Detection | Dual |
| 79 | CN219855268 | 2022 | China | TL/Towers | AI/CV | Physical Barriers | Detection + Mitigation | Dual |
| 80 | CN219877280 | 2022 | China | TL/Towers | Passive | Physical Barriers | Mitigation | General |
| 81 | CN220192898 | 2022 | China | TL/Towers | Passive | Physical Barriers | Mitigation | General |
| 82 | CN220586975 | 2022 | China | TL/Towers | Passive | Physical Barriers | Mitigation | General |
| 83 | CN220630787 | 2022 | China | TL/Towers | Passive | Physical Barriers | Mitigation | General |
| 84 | CN220630786 | 2022 | China | TL/Towers | AI/CV | Physical Barriers | Detection | General |
| 85 | IN202311074124 | 2023 | India | TL/Towers | AI/CV | Physical Barriers | Detection | Dual |
| 86 | CN217446382 | 2022 | China | TL/Towers | Passive | Physical Barriers | Mitigation | General |

# Table S2. Market analysis of bird detection and mitigation technologies (2024–2030)

**Note:** *Market estimates and projections are derived from non-peer-reviewed industry consultancy reports. Substantial methodological variability exists across sources, reflecting differences in market segmentation, geographic scope, and technological scope definitions. All monetary values are in USD millions. CAGR = compound annual growth rate (%). These figures should be interpreted as indicative trends rather than precise empirical measurements and should not be cited as primary evidence in scientific analyses.*

| Source | Market Value 2024 (USD M) | Projected Value 2030 (USD M) | CAGR (%) |
| --- | --- | --- | --- |
| Future Market Insights (2024) | ~140.00 | ~190.12 | 5.0 |
| Business Research Insights (2024) | 130.68 | 231.42 | 10.0 |
| Stratview Research (2024) | 140.86 | 224.37 | 8.0 |
| Global Growth Insights (2024) | 120.06 | 236.57 | 11.2 |
| Average | **132.90** | **220.62** | **8.6** |

**References (Supplementary Material)**

Business Research Insights. (2024). Bird detection system market: Global industry analysis, market size, trends, growth and forecast 2024–2030. Industry report.

Future Market Insights. (2024). Bird detection system for airports market: Global industry analysis and opportunity assessment 2024–2034. Industry report.

Global Growth Insights. (2024). Bird detection systems market: Industry trends, growth, and forecast analysis. Industry report.

Stratview Research. (2024). Bird detection systems market report: Trends, forecast, and competitive analysis 2024–2030. Industry report.
